# Predicting Trustworthiness Across Cultures: An Experiment

**DOI:** 10.3389/fpsyg.2021.727550

**Published:** 2021-09-28

**Authors:** Adam Zylbersztejn, Zakaria Babutsidze, Nobuyuki Hanaki

**Affiliations:** ^1^Univ Lyon 2, Université Lumière Lyon 2, GATE L-SE UMR 5824, Lyon, France; ^2^Vistula University Warsaw (AFiBV), Warsaw, Poland; ^3^SKEMA Business School, Université Côte d'Azur (GREDEG), Valbonne, France; ^4^Observatoire Français des Conjonctures Economiques (OFCE), Sciences Po, Paris, France; ^5^Institute of Social and Economic Research, Osaka University, Osaka, Japan

**Keywords:** trustworthiness, communication, hidden action game, cross-cultural comparison, laboratory experiment

## Abstract

We contribute to the ongoing debate in the psychological literature on the role of “thin slices” of observable information in predicting others' social behavior, and its generalizability to cross-cultural interactions. We experimentally assess the degree to which subjects, drawn from culturally different populations (France and Japan), are able to predict strangers' trustworthiness based on a set of visual stimuli (mugshot pictures, neutral videos, loaded videos, all recorded in an additional French sample) under varying cultural distance to the target agent in the recording. Our main finding is that cultural distance is not detrimental for predicting trustworthiness in strangers, but that it may affect the perception of different components of communication in social interactions.

## 1. Introduction

A common pattern in human strategic behavior is conditional cooperation, i.e., the willingness to sacrifice personal resources for the mutual benefit as long as others do the same (Fischbacher et al., 2001; Kocher et al., 2008). The extent to which individuals follow the notion of conditional cooperation determines their trustworthiness in social interactions that require mutual cooperation or involve economic exchange (Boone and Buck, 2003). Notwithstanding the standard economic prediction that communication in such contexts should be “cheap talk” and considered as irrelevant for final decisions (Farrell and Rabin, 1996), but in line with the “mind reading” hypothesis that communication may help uncover the motivational states of others (Sally, 2000), experimental evidence suggests that communication helps detect trustworthiness. Communication can thus contribute to creating successful partnerships, and help protect against potential exploitation (He et al., 2017).

Clearly, the verbal content of communication may provide valid signals for the receiver about the sender's intentions. A well-established finding is that making a voluntary promise (i.e., a free statement of intent) to cooperate is predictive of the sender's cooperative behavior (see Woike and Kanngiesser, 2019, for a recent and exhaustive review of this vast literature). In addition, Babutsidze et al. (2021) provide experimental evidence that this signal is correctly taken into account by the receivers across several communication protocols (ranging from plain text transcript to audio recording to video recording to face-to-face interaction) varying the amount of nonverbal content conveyed in the sender's message.

However, communication in social interactions is not only about words. Under the standard definition applied in animal studies, communication consists of any *behavior in [*…*] the sender [*…*] which evokes a response in [*…*] the receiver*; for humans, this definition may also encompass notions of conscious intent or volition (see Chapter 2 in Ekman, 2006, p. 21). Accordingly, another important result in the experimental literature is that the role of communication as means of signaling trustworthiness is not restricted to its purely verbal content. The nonverbal components of communication—such as facial displays, body movements, tone of voice—also play a role in signaling trustworthiness. For instance, echoing the evolutionary argument by Boone and Buck (2003) that spontaneous emotional expressivity can act as a marker of pro-social motives like trustworthiness and cooperativeness, Brown et al. (2003) provide experimental evidence that altruists are perceived as more expressive than non-altruists. Oda et al. (2009b) highlight a particular dimension of human emotional expressivity: altruists are more likely to display genuine smiles. In the same vein, Centorrino et al. (2015) investigate the role of smiles in creating social exchange. Using an incentivized trust game with pre-play communication stage in which the trustee transmits to the trustor a pre-recorded video message with standardized verbal content, they find that the trustees conveying genuine smiles in their recordings also tend to be more trustworthy (i.e., generous toward their partners), and incite higher trust from others. An important line of experimental work also shows that information gathered through a brief, controlled and superficial access to physical characteristics of an unknown counterpart—their face, body gestures, way of expression (sometimes referred to as “thin slices” of observable information)—may help detect cooperativeness in various types of economic interactions (for a recent survey, see Bonnefon et al., 2017).

Our paper contributes to the growing experimental literature on detecting other-regarding preferences based on “thin slices” of observable information. We investigate the extent to which the recognition of trustworthiness in social interactions is a pancultural trait. We address the following question: Does cultural distance matter when it comes to detecting trustworthiness in social interactions? We build on a series of previous experiments by Oda et al. (2009a) and Tognetti et al. (2018) who offer a cross-cultural (Japan vs. France) comparison of the ability to detect the degree of altruism of Japanese subjects based on a short and muted video recording taken in a context which is unrelated to the target behavior. Tognetti et al. (2018) interpret the main finding—the general capacity (inability) of the Japanese (French) subjects to distinguish between altruistic and non-altruistic Japanese subjects based on the provided visual stimuli—as evidence that the nonverbal cues of prosociality are specific to one's culture rather than universally detectable. Our laboratory experiment is based on a variation of the trust game (Berg et al., 1995) with moral hazard, known as the hidden-action game (Charness and Dufwenberg, 2006). Our first set of stimuli comes from the previous experimental dataset reported by Babutsidze et al. (2021). It consists of video recordings of short, free-form pre-play statements delivered by the trustees to the trustors in direct face-to-face interactions happening in Nice, France. We provide the nonverbal content of those recordings as stimuli in an incentivized task in which subjects need to correctly predict the decisions previously made by the trustees. To allow for a cross-cultural comparison of prediction accuracy, this part of experiment relies on a different French sample (Lyon), as well as on a Japanese sample (Osaka).

As compared to the standard prediction tasks employing the “thin slice” paradigm, our methodological focus on nonverbal communication is novel and taps into the behavioral ecology of laboratory experimentation with social interactions. From the behavioral ecology perspective, facial displays are specific to intent and context, are issued in the service of social motives, and are interpretable in the context of interaction (see, e.g., Chapter 7 in Fridlund, 1994). In the words of Chovil and Fridlund (1991):

*Facial displays are a means by which we communicate with others. Like words and utterances, they are more likely to be emitted when there is a potential recipient, when they are useful in conveying the particular information, and when that information is pertinent or appropriate to the social interaction*. (p. 163)

Clearly, this argument also applies to other components of nonverbal communication, such as gestures and body language. However, the previous studies—including those mentioned above (the study by Centorrino et al., 2015, is a notable exception), as well as the later contributions by, e.g., Van Leeuwen et al. (2018) and Oda et al. (2021)—are typically based on visual stimuli which are strongly dissociated from the social context in which the predicted target behavior (i.e., detection of certain facets of cooperativeness, such as altruism, trustworthiness, reciprocity) occurs. This is either because the visual stimuli used therein only consist of a neutral mugshot picture (like in our first control condition—PHOTO) or a neutral video recording with made-up content (like in our second control condition—neutral video, henceforth VIDNE)^1^. Thus, such standard design may only capture the extent to which certain morphological characteristics and general expressivity can be helpful in predicting human behavior. Our main condition (loaded video, henceforth VIDLO) extends this standard setup by providing the visual stimuli that belongs to the same social context as, and thus is intertwined with, the target behavior—the personal statement made by a trustee in front of the trustor prior to the decision-making stage of the trust game. Thus, the “thin slice” of observable information and the subsequent target behavior are both components of the same social interaction^2^.

We find several consistent patterns of prediction-making in our two samples. For both samples, the overall rates of accurate detection of trustworthiness in strangers based on “thin slices” of observable information remain constant across the three types of stimuli. Moreover, we look at certain morphological traits of the target agents (facial masculinity, asymmetry, and weight-to-height ratio, as well as sex) and find that both the French and the Japanese subjects resort to the same heuristics (thus exhibiting similar biases) when making judgments about others' trustworthiness.

Nonetheless, some notable differences also arise across the two cultures. Overall, the VIDLO condition is the only instance where we observe predictions being made with a “better than chance” accuracy. However, this only happens for the Japanese subjects; despite cultural proximity with the target agents, the French subjects are not able to distinguish between the trustworthy and untrustworthy ones after observing the nonverbal content of communication. To shed more light on this (somewhat surprising) outcome, we then extend our empirical analysis with a new dataset containing the same set of recordings, this time with unmuted verbal content. The availability of this verbal content significantly improves prediction accuracy of the French subjects in the unmuted VIDLO condition. In line with the previous studies, we confirm a particular role of voluntary promises in signaling trustworthiness among strangers. This suggests that cultural distance (proximity) makes people relatively sensitive (insensitive) to the relevant components of nonverbal content of communication that go beyond basic morphological heuristics. Rather, within cultural proximity attention is attuned to the relevant aspects of the verbal content of communication. Hence, cultural distance *(i)* is not detrimental for the comprehension of the nonverbal content of communication (if anything, it is exactly the opposite), and *(ii)* it may affect the perception of the different components of communication in social interactions.

## 2. Experimental Design

### 2.1. Experimental Stimuli for the Prediction Task

**Figure 1:**
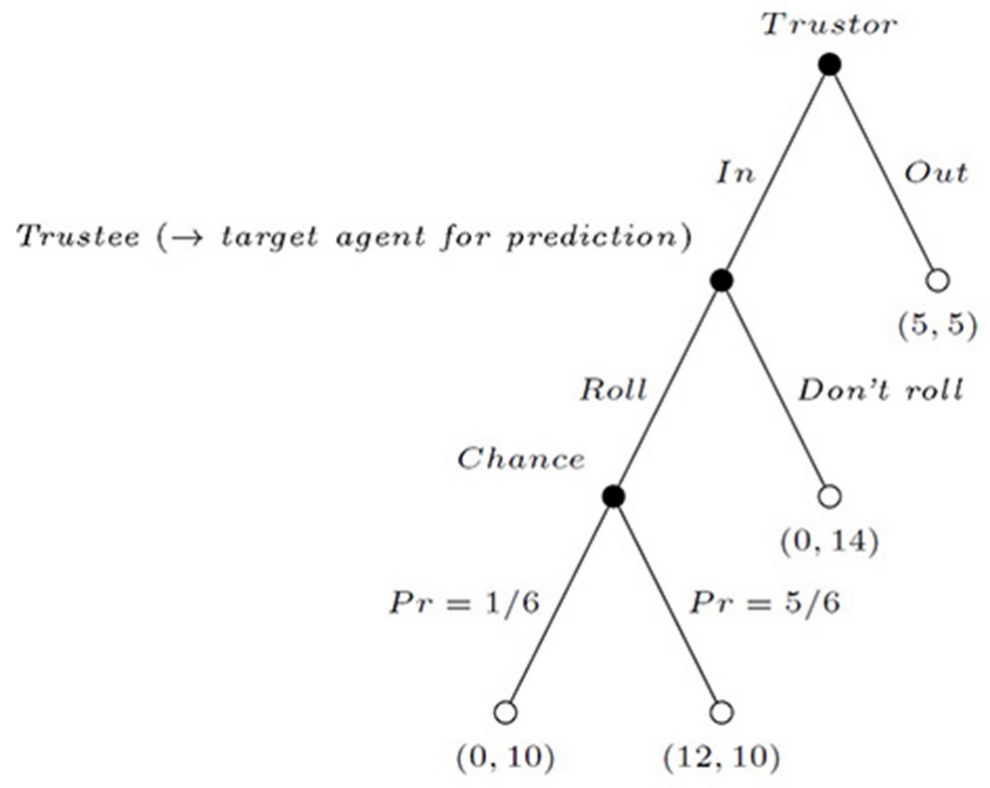
Experimental hidden action game.

For implementing the prediction task, we exploit the dataset previously reported in Babutsidze et al. (2021). That study is based on the hidden action game by Charness and Dufwenberg (2006) presented in Figure 1. All payoffs are in Euros. The game is played between two parties: the trustor and the trustee. The trustor may either choose an outside option *Out* which yields 5 to both players and ends the interaction, or go *In*. Then, the trustee may either choose to *Roll* a die (which yields 12 to the trustor and 10 to the trustee with the probability of 5/6, and 0 to the trustor and 10 to the trustee with the probability of 1/6), or not to *Roll* (yielding 0 to the trustor and 14 to the trustee with certainty). This game provides a simple setting for studying voluntary cooperation under moral hazard: incentives are not aligned between the two parties, and earning 0 is not perfectly informative for the trustor about the trustee's action. For this reason, we believe that the hidden action game offers a conservative way of measuring trustworthiness compared to the classic trust game due to Berg et al. (1995).

Like Charness and Dufwenberg (2006), we simultaneously elicit both players' decisions. Namely, the trustee makes a decision without knowing the trustor's move, and that decision is only implemented had the trustor gone *In*. The game is preceded by a pre-play stage with face-to-face communication and is implemented as follows. In every experimental session, six trustors are seated in one room (in separate cubicles and without the possibility to communicate) where they make all their decisions in the game. Each of the six trustees, in turn, makes an individual decision in a separate room. Prior to the decision-making stage of the game, each trustee is given approximately two minutes to prepare a short statement for the trustors. At this point, we provide an additional set of instructions emphasizing the fact that the statement may affect the trustors' decisions and, consequently, the trustee's gain from the experiment^3^. Then, the trustee enters the trustors' room and delivers the statement in front of them. The trustors can clearly see and hear the trustee, and the trustee can also observe the trustors while delivering the statement. After that, the trustee leaves to a separate room to make a decision. Simultaneously, the six trustors privately make their decisions. At the end of the experiment, the trustees and the trustors are randomly and anonymously matched into six pairs for payments. Further implementation details, including the instructions used in that experiment, are provided in Appendices A1, A2.

In addition to the trustees' decisions in the experimental game (and, if relevant, the outcomes of die rolls), our dataset contains several recordings. Following Van Leeuwen et al. (2018), upon arrival to the laboratory and before learning about the rules of the hidden action game, each subject in the role of a trustee is invited to a separate room for a mugshot picture and a standardized video recording: the subjects are asked to read a short extract from a printer instruction manual, while keeping a neutral facial expression. These two sources of information are used, respectively, in our PHOTO and VIDNE (neutral video) treatments. Finally, the trustees are also video recorded while making a statement in the pre-play communication stage of the hidden action game. We use this information in our VIDLO (loaded video) treatment.

The original database in Babutsidze et al. (2021) includes 41 trustees and has been collected at Laboratoire d'Economie Expérimentale de Nice (LEEN) of the University of Nice, France. These participants gave their explicit consent (*i*) for being recorded, and (*ii*) for those recordings being used for strictly scientific purposes in related experimental studies. For the sake of the present study, we restrict the set of stimuli to an ethnically homogeneous group of subjects classified as Caucasian by an independent coder (*N* = 26; 13 females; average age 22.58, SD = 3.18). Furthermore, we do not disclose the location in which this sample was collected. The purpose of these design choices is to minimize the role of ethnic and/or racial biases in reaction to each stimulus. These trustees are the target agents in the prediction tasks implemented in the main experiment. Among these 26 target agents, 16 chose to *Roll*. The 26 stimuli are presented in random order.

### 2.2. Main Experiment

Our main experiment is implemented through a between-subject design and involves a total of *N* = 273 participants (97% students; 53% Japanese; 40% females; average age 21.51, SD = 3.89). Table 1 provides further information about the assignment of subjects in our 3 × 2 factorial design: across the three treatments (PHOTO, VIDNE, VIDLO) and two locations (Lyon, France and Osaka, Japan). For each of the six conditions, we run two experimental sessions that took part in May 2018 in the Experimental Economics Laboratory at the Institute of Social and Economic Research (ISER) at Osaka University in Japan, and in December 2019 in the GATE-Lab, an experimental laboratory at the GATE Lyon-Saint-Etienne research institute in France^4^. Experimental sessions were entirely computerized: subjects were recruited using ORSEE (Greiner, 2015), and all the experimental tasks were programmed in z-Tree (Fischbacher, 2007).

**Table 1:** Average prediction accuracy rates across countries and treatments: aggregate data.

	**France**	**Japan**	***p***
PHOTO	51.0% (*N* = 43)	50.9% (*N* = 50)	0.972
VIDNE	52.1% (*N* = 37)	51.6% (*N* = 49)	0.814
VIDLO	49.9% (*N* = 48)	52.3% (*N* = 46)	0.209
*p*	0.533	0.779	

*p-values in the last column (row) come from a two-sided t-test (F-test) of the equality of prediction accuracy rates between countries for a given treatment (across treatments within a given country)*.

Participants make a series of twenty six predictions of trustees' behavior in an earlier hidden action game (i.e., whether the target person rolled a die or not). A correct (an incorrect) prediction is worth 10 (2) euros in the experiments run in France, and 1,200 (240) yen for those run in Japan. No feedback is provided from one prediction to the other, and two rounds out of twenty six are randomly drawn for payoff at the end of each experimental session. Unlike some previous studies using the “better than chance” paradigm, we do not constrain the base rate of “success” at the chance level of 50%^5^. Our experimental treatments progressively enrich the set of information about the trustee that is provided to the subject prior to making a prediction: either a mugshot picture (PHOTO), or one of muted video recording: either showing that person making a non-strategic statement that has been recorded before (and independently of) the experimental hidden action game (VIDNE), or a loaded one in which the trustee makes a strategic pre-play statement in front of the trustors (VIDLO)^6^.

### 2.3. Experimental Procedures

Upon arriving to the lab, subjects are seated in individual cubicles and informed about the general rules of a lab experiment^7^. The preliminary part of the session consists of a basic socio-demographic questionnaire (age, sex, education, major, current occupation, score at the *baccalauréat* exam at the end of high school in the case of French subjects), as well as a set of (moderately) incentivized and non-incentivized computerized tasks designed to measure specific individual characteristics^8^. After that, subjects receive paper instructions describing the details of the previous hidden action game experiment, as well as their own experimental task.

Those instructions are read aloud by the experimenter, any remaining questions are immediately answered, and the experiment moves to its main stage, as described above. In addition to earnings in the experimental tasks, there is a show-up fee of 5 euros for the French participants, and 600 yen for the Japanese participants. The duration of a session was approximately 1h30 and the average total payoff was 23 euros in France and 3,175 yen in Japan^9^.

## 3. Aggregate Results

Table 1 provides an overview of the average prediction accuracy rates (i.e., the likelihood that a randomly chosen subject makes a correct prediction in a randomly chosen round of the experiment) across treatments and cultures. This aggregate evidence points to *(i)* no effects of varying the sources of observable information on prediction accuracy within a given culture, and *(ii)* no intercultural variation of prediction accuracy in any of the three information conditions.

As a next step of our analyses, we disaggregate those data by looking at prediction accuracy rates conditional on the target agent's actual decision—either *Roll* or *Don't roll*. We employ the statistical framework from Zylbersztejn et al. (2020) to draw a link between the predicted behavior and the actual behavior. Suppose that *p*_*R*_ (*p*_*DR*_) is the probability of making a prediction *Roll* conditional on the target person actually choosing to *Roll* (*Don't roll*). *p*_*R*_ = *p*_*DR*_ implies that subjects are unable to discriminate between trustworthy and untrustworthy target players, and make a prediction *Roll* at a constant rate (freely ranging between 0 and 1) irrespective of the trustee's underlying type. *p*_*R*_ > *p*_*DR*_, in turn, implies that subjects are able to detect the target player's type at least partially which makes them more likely to make a prediction *Roll* for those who actually rolled a die^10^. The corresponding prediction rates are summarized in Table 2, and statistical support for mean comparisons is provided in Table 3. For each of the three information conditions (PHOTO, VIDNE, VIDLO), we regress an indicator variable 1[*PredictionRoll*] (set to 1 if one predicts that the target person rolled a die in the previous experiment, and to 0 otherwise) on another indicator variable 1[*ActualRoll*] (set to 1 if the target person actually rolled a die in the previous experiment, and to 0 otherwise), 1[*Japan*] (set to 1 for the Japanese subjects, and to 0 otherwise), as well as their interaction. The intercept (denoted α_0_) captures the aggregate likelihood of predicting *Roll* for those trustees that did not actually roll a die (such that α_0_ = *p*_*DR*_). Our key measure of interest is given by coefficients α_1_ and α_1_ + α_3_ which provide the respective empirical estimates of the difference between *p*_*R*_ and *p*_*DR*_ (i.e., the extent to which subjects are able to distinguish between those who rolled and those who did not) for the French and Japanese subjects^11^.

**Table 2:** Predicted vs. actual behavior: prediction accuracy across countries and treatments.

	***Pr*(1[***PredictionRoll***]) = 1**			
**If 1[*ActualRoll*] =**	**0**	**1**	**0**	**1**
	**(*p*_*DR*_)**	**(*p*_*R*_)**	**(*p*_*DR*_)**	**(*p*_*R*_)**
**Condition**	**France**		**Japan**	
PHOTO	44.2%	46.8%	38.2%	41.6%
VIDNE	45.3%	49.8%	42.5%	46.5%
VIDLO	50.0%	49.9%	36.2%	42.4%

*1[PredictionRoll] (1[ActualRoll]) is set to 1 if a subject predicts that the target player rolled a die (if the target player actually rolled a die) in the previous experiment, and to 0 otherwise*.

**Table 3:** Predicted vs. actual behavior: regression analysis.

	**PHOTO**		**VIDNE**		**VIDLO**	
	**Coef**.	**p**	**Coef**.	**p**	**Coef**.	**p**
	**(SE)**		**(SE)**		**(SE)**	
Intercept (α_0_)	0.442	<0.000	0.453	<0.000	0.500	<0.000
	(0.042)		(0.031)		(0.025)	
1[*ActualRoll*] (α_1_)	0.027	0.212	0.045	0.162	−0.001	0.955
	(0.021)		(0.032)		(0.026)	
1[*Japan*] (α_2_)	−0.060	0.267	−0.028	0.535	−0.138	0.002
	(0.054)		(0.044)		(0.044)	
1[*ActualRoll*] × 1[*Japan*] (α_3_)	0.007	0.816	−0.006	0.895	0.063	0.086
	(0.032)		(0.042)		(0.036)	
*H*_0_ : α_1_ + α_3_ = 0		0.159		0.134		0.016
*Prob* > *F*		0.172		0.171		0.005
*N* of obs./clusters		2418/93		2236/86		2444/94

*Results of OLS regression models of the individual prediction (indicator variable 1[PredictionRoll] = 1 if one predicts that the target player rolled a die in the previous experiment; 0 otherwise) on a set of indicator variables: 1[ActualRoll] (set to 1 if the target player actually rolled a die in the previous experiment, and to 0 otherwise), 1[Japan] (set to 1 for the Japanese subjects, and to 0 otherwise), as well as their interaction. Observations are clustered for each individual, standard errors (SE) are cluster-robust*.

The main message that stems from this analysis is the following: only in one instance—the VIDLO condition implemented in Japan—the difference *p*_*R*_ − *p*_*DR*_ is positive and statistically significant (testing *H*_0_ : α_1_ + α_3_ = 0 yields *p* = 0.013), indicating that these subjects can tell better than chance between trustworthy and untrustworthy target agents. In the five remaining cases, we observe *p*_*R*_ − *p*_*DR*_ to be small and not significantly different from zero^12^.

### 3.1. The Role of Target Player's Facial Characteristics

The model reported in Table 4 extends the analyses from Table 3 by accounting for several individual characteristics of the target player. Beside the treatment and 1[*ActualRoll*] indicator variables, as well as their interactions (coefficients β_1_, …, β_5_), the set of explanatory variables includes several facial measurements of the target agent (masculinity, asymmetry, weight-to-height ratio; coefficients β_6_, β_7_, β_8_, respectively) and that person's sex (1[*Female*] = 1 for females, 0 for males; coefficient β_9_)^13^. Furthermore, we include an indicator variable 1[*Japan*] (set to 1 for the Japanese subjects and to 0 otherwise; coefficient γ_0_) and its interactions with all the previous variables (coefficients γ_1_, …, γ_9_). The model is estimated with pooled data^14^.

**Table 4:** Facial characteristics and predictions across cultures: regression analysis.

**Coef. number (i): Variable**	**β_*i*_**	**p**	**γ_*i*_**	**p**
	**(SE)**		**(SE)**	
0: Intercept	0.312	0.005	0.096	0.513
	(0.110)		(0.147)	
1: 1[*ActualRoll*]	0.019	0.363	0.014	0.671
	(0.021)		(0.032)	
2: 1[*VIDNE*]	0.011	0.836	0.033	0.639
	(0.052)		(0.069)	
3: 1[*VIDLO*]	0.058	0.237	−0.077	0.263
	(0.049)		(0.069)	
4: 1[*ActualRoll*] × 1[*VIDNE*]	0.019	0.625	−0.013	0.804
	(0.038)		(0.052)	
5: 1[*ActualRoll*] × 1[*VIDLO*]	−0.028	0.403	0.056	0.250
	(0.034)		(0.048)	
**Target agent's characteristics**				
6: Facial masculinity	0.018	<0.000	0.007	0.219
	(0.004)		(0.006)	
7: Facial asymmetry	0.003	0.292	−0.004	0.212
	(0.003)		(0.003)	
8: Facial width-to-height ratio	0.002	0.970	−0.076	0.183
	(0.042)		(0.057)	
9: 1[*Female*]	0.087	<0.000	0.007	0.822
	(0.022)		(0.030)	

*Results of OLS regression models of the individual prediction (indicator variable 1[PredictionRoll] = 1 if a subject predicts that the target agent rolled a die in the previous experiment; 0 otherwise) on a set of explanatory variables: 1[ActualRoll] (set to 1 if the target agent actually rolled a die in the previous experiment, and to 0 otherwise) and treatment indicator variables 1[VIDNE] and 1[VIDLO] set to 1 for a given treatment and 0 otherwise (1[PHOTO] is the omitted reference condition), as well as their interactions; target player's individual characteristics: facial masculinity, facial asymmetry, facial weight-to-height ratio, as well as sex (1[Female] is set to 1 for females, and to 0 for males). This subset of explanatory variables is associated with coefficients β_i_ (first column). The model also includes an indicator variable 1[Japan] (set to 1 for the Japanese subjects, and to 0 otherwise) as well as its interactions with all the previous variables; these explanatory variables are associated with coefficients γ_i_ (last column). Observations are clustered for each individual (7,098 observations in 273 clusters), standard errors (SE) are cluster-robust*.

This new specification *(i)* provides robustness analysis of the effects reported in Table 3 after controlling for a rich set of target player's observable characteristics, and *(ii)* allows for testing (through coefficients γ_*i*_) for cultural differences with respect to any of the dimensions captured by the model.

In relation to *(i)*, the model confirms that only in one instance—the VIDLO condition implemented in Japan—relevant information can be extracted from the recordings in a way that improves prediction accuracy above chance^15^.

Regarding *(ii)*, the model indicates that, irrespective of the culture of origin, subjects systematically condition their predictions on certain observable characteristics of the target players. It is important to note at this point that, based on our empirical data, this information should be considered as irrelevant for predictions, since neither of the four individual characteristic included in the model happens to be associated with the observed behavior in the hidden action game^16^. Nonetheless, two of these observable characteristics—facial masculinity and sex—are statistically significant predictors of assessed trustworthiness. Importantly, such biased judgment of trustworthiness persists across cultures^17^.

### 3.2. The Role of Verbal Content

So far, our experimental evidence points to a general incapacity of the French subjects to accurately predict strangers' trustworthiness from different stimuli containing nonverbal content, despite cultural proximity between the two parties. Strikingly, this failure occurs even for the strategically loaded video recordings provided in the VIDLO condition—stimuli that helps the more culturally distant Japanese subjects distinguish between the target players' types. In this section, we are asking whether and to what extent this insufficiency can be fixed by further providing the verbal content of VIDLO recordings. For this sake, we revisit the dataset from our previous experiment reported in Zylbersztejn et al. (2020). That experiment involves the same subject pool (GATE-Lab, Lyon, France) and the same video recordings, but this time with sound turned on (henceforth referred to as the VIDLO_SOUND condition)^18^.

Evidence reported in the first part of Table 5 suggests that, unlike the sound-off VIDLO condition, the VIDLO_SOUND condition with verbal content of strategic statements allows the French subjects to distinguish between the target agents' types. Even though the ability to identify untrustworthy target players does not vary between the two conditions, we observe that VIDLO_SOUND improves detection of trustworthiness. Furthermore, in line with a large body of experimental literature (see Woike and Kanngiesser, 2019, for a recent review), these data indicate that a particular facet of verbal content—a promise to *Roll*—constitutes an informative signal of cooperative intentions: target agents who made such a promise are more than twice as likely to *Roll* than the target players not making such a promise^19^.

**Table 5:** Verbal and nonverbal content in VIDLO: evidence from the French data.

**Average rate of prediction** ***Roll*** **per stimulus**			
**If 1[*ActualRoll*] =**	**0 (*N* = 12)**	**1 (*N* = 14)**	**p (ranksum test)**
VIDLO_SOUND	47.9%	66.2%	0.024
VIDLO	50.0%	49.9%	0.918
*p* (signrank test)	0.814	0.035	
**If 1[*PromiseRoll*] =**	**0 (*N* = 10)**	**1 (*N* = 16)**	**p (ranksum test)**
VIDLO_SOUND	47.6%	64.1%	0.045
VIDLO	54.8%	46.9%	0.119
*p* (signrank test)	0.445	0.015	

*The unit of observation is the rate of prediction Roll observed for a given recording (N = 26) in a given condition. 1[ActualRoll] (1[PromiseRoll]) is set to 1 if the target player actually rolled a die (made a promise to roll a die) in the previous experiment, and to 0 otherwise*.

As shown in the bottom part of Table 5, French subjects in the VIDLO_SOUND condition effectively pick up on this signal and attribute higher trustworthiness to promise-makers, in stark contrast to the sound-off VIDLO condition. We also note that the same holds for the Japanese sample: the respective rates are 48.2% without a promise, and 37.5% with a promise (*p* = 0.118, two-sided ranksum test). This, in turn, suggests that the nonverbal information the Japanese subjects pick up on when forming judgments is unrelated to the verbal content conveyed in the strategic statements^20^.

## 4. Conclusion

Our study contributes to several strands of ongoing debate on how observing others may be helpful for predicting their behavior in social interactions. We take a cross-cultural perspective and focus on the ability to detect a stranger's proneness to conditional cooperation, or trustworthiness, based on “thin slices” of observable information. As noted by Olivola et al. (2014), many important social decisions (e.g., political elections and court sentences) are made on the basis of people's facial appearance, and individuals tend to agree when it comes to judging which faces look trustworthy^21^. Furthermore, evidence from laboratory experiments employing economic games suggests that people exhibit less trust toward partners with untrustworthy looking faces, even when given relevant information about their past behavior (Chang et al., 2010; Rezlescu et al., 2012).

Is this information actually useful for making accurate judgments? Olivola et al. (2014) and Todorov et al. (2015a) qualify “face-ism” as a judgment bias, since social inferences based on facial appearance tend to be inaccurate and unreliable. On the other hand, Bonnefon et al. (2013, 2017) argue that physical cues provided via “thin slices” of information may nonetheless contain “kernels of truth,” and observing one's face, body language, way of expression may help detect cooperation in various economic interactions.

We believe that our novel experimental evidence goes some way in reconciling both of these claims. Echoing a closely related study by Tognetti et al. (2013), our experimental data point to a judgment bias that meshes well with the notion of “face-ism”: subjects account for morphological traits of the target agents, even though the latter are not associated with the actual behavior. Extending these previous findings, we further document that this bias persists across cultures and attains the same magnitude in both the French and the Japanese sample.

At the same time, we believe that “kernels of truth” may well exist alongside the aforementioned biased judgments. However, our data reveals that predicting behavior in social interactions requires that “thin slices” contain direct social cues (like in our VIDLO condition), rather than being restricted to the purely physical ones (i.e., with no relation to the social context of the interaction—like in our PHOTO and VIDNE conditions). The dominant role of social context relative to physical attributes is consistent with a recent study by Jaeger et al. (2020) who show that people are generally unable to detect the trustworthiness of strangers based solely on their facial appearance. Importantly, we find that this effect varies considerably across cultures. Despite cultural distance, Japanese subjects are sufficiently attuned to the nonverbal content of strategic statements to be able to distinguish between trustworthy and untrustworthy target agents in the VIDLO condition. Within cultural proximity, French subjects tend to ignore these cues. Nonetheless, when additionally provided with verbal content (like in our auxiliary VIDLO_SOUND condition), they become capable of correctly reading a credible signal of trustworthiness—namely, a voluntary promise to cooperate. Hence, we conclude that cultural distance is not *per se* helpful or detrimental for predicting trustworthiness. Rather, it affects ways in which people exploit observable information in social interactions.

In the closing lines, we would like to mention an important limitation of our study. Both the target agents used in the experimental stimuli, as well as the sample of participants to our experiment, are drawn from rather homogeneous student populations in France and Japan. While we see our study as an important step in documenting cross-cultural differences in trustworthiness detection, we also believe that there is a need for further evidence drawn from different sets of stimuli (e.g., including ethnicities other than the Caucasian ethnicity we focus on here) and more diversified samples of participants (e.g., coming from the general population).

## Data Availability Statement

The raw data supporting the conclusions of this article will be made available by the authors, without undue reservation.

## Ethics Statement

The studies involving human participants were reviewed and approved by the GATE-Lab Research Ethics Committee based at the Groupe d'Analyse et de Théorie Economique (UMR 5824). The patients/participants provided their written informed consent to participate in this study.

## Author Contributions

All authors listed have made a substantial, direct and intellectual contribution to the work, and approved it for publication.

## Funding

We acknowledge the support from the following programs operated by the French National Research Agency (Agence Nationale de Recherche): DigiCom as a part of *UCA*^*JEDI*^ (ANR-15-IDEX-01) and LABEX CORTEX (ANR-11-LABX-0042) as a part of Université de Lyon (ANR-11-IDEX-007), as well as the Joint Usage/Research Center at ISER, Osaka University, and Grant-in-aid for Scientific Research, Japan Society for the Promotion of Science (15H05728, 18K19954, 20H05631).

## Conflict of Interest

The authors declare that the research was conducted in the absence of any commercial or financial relationships that could be construed as a potential conflict of interest.

## Publisher's Note

All claims expressed in this article are solely those of the authors and do not necessarily represent those of their affiliated organizations, or those of the publisher, the editors and the reviewers. Any product that may be evaluated in this article, or claim that may be made by its manufacturer, is not guaranteed or endorsed by the publisher.

## Footnotes

^1^These two sets of stimuli come from our previous experimental work reported in Zylbersztejn et al. (2020) and Babutsidze et al. (2021).

^2^For a similar approach based on non-experimental data see, e.g., Belot et al. (2010, 2012), Sylwester et al. (2012), Van den Assem et al. (2012), Turmunkh et al. (2019). They use data from a TV game show—*The Golden Balls*—which consists of a high stake prisoner's dilemma environment with a pre-play stage of natural face-to-face communication moderated by the host. Despite the clear virtues in terms of behavioral ecology, some features of these data fall short of the rigorous requirements of experimental control that is achieved in our laboratory setting. First, there is a continuous two-way communication between participants, so each subject acts both a sender and a receiver of messages. In our design, the players' roles in the process of communication are unique and reflect their respective tasks in the game. Second, in a TV game show the process of communication may be interrupted, and its content affected by a third party: the game host. For instance, often times the host talks one player into making a promise to cooperate with the other player. Our design rules our any possibility of such interference, allowing for a free and uninterrupted flow of communication from the trustee to the trustor.

^3^This information is part of the summary of the hidden action game experiment provided in the instructions employed in the current study.

^4^Since acquaintance between the experimental subjects in Lyon and the target agents recorded in Nice is unlikely, one may plausibly assume that performance in the prediction task actually measures the individual capacity to detect cooperativeness in strangers. See Centorrino et al. (2015) and Van Leeuwen et al. (2018) for a similar approach.

^5^Under the “better than chance” paradigm, subjects typically receive randomly generated pairs of stimuli—one coming from a person that exhibited certain behavior, and one from another person that did not (which is common knowledge; see, e.g., Bonnefon et al., 2013; Van Leeuwen et al., 2018). Another method is to show a series of individual stimuli and inform the subjects about the underlying base rate (50%) of a given behavioral outcome, but not about the length of the series (Vogt et al., 2013). Although the “better than chance” paradigm provides a clean and simple benchmark for measuring the extent to which observable information affects prediction accuracy, it has been criticized for the lack of external validity. As pointed out by Todorov et al. (2015a), this criterion seems weak when it comes to evaluating prediction performance in many real-world environments in which the different types of behavior are unequally prevalent. Following this argument, in our experiment the lack of information about the underlying base rate adds to the overall complexity of the prediction task. See Fetchenhauer et al. (2010) for a similar approach.

^6^The average duration of a recording in VIDNE (VIDLO) is 33.38 (25.85) s with SD 5.27 (13.31) and range 27–49 (11–60). Given that PHOTO only involves static content, in this treatment we adopted the following procedure. Each time, the picture of the target person is displayed on the computer screen. After 15 s, a button appears underneath the picture allowing the subject to move on to the prediction-making stage. This choice came about as the outcome of the pilot test of our experimental setup, and appears to be a remedy against the risk of “under-exposing”—the exposure to the displayed content being insufficient to fully grasp all the available information, as well as “over-exposing”—participants eventually getting inattentive due to factors such as boredom, impatience, or a decay in their interest in the displayed static content.

^7^The original instructions are in French for the experiments run in Lyon, and in Japanese for those run in Osaka. Their English version can be found in Appendix A3.

^8^This procedure closely follows Babutsidze et al. (2021), and its details can be found therein. The set of tasks includes standard measures of other-regarding preferences (Social Value Orientation, SVO, task by Murphy et al., 2011), cognitive skills (3-item Cognitive Reflection Test, CRT, Frederick, 2005), the theory of mind (The Reading the Mind in the Eyes Test, RMET, Baron-Cohen et al., 2001), risk preferences (Gneezy and Potters, 1997), and general trust attitudes (based on the German Socio-Economic Panel Study, SOEP). In most cases, we find no differences between the two samples—this applies to distributional preferences, cognitive skills, risk preferences, and general attitudinal trust toward other people. One notable exception, however, is the theory of mind: the French subjects attain a significantly higher score on RMET (mean scores of out 34: 27.28 vs. 21.71, *p* < 0.001 based on two-sided *t*-test). However, in neither experimental environment of our 3 × 2 experimental design we observe statistically significant (Spearman's rank) correlation between this measure of the theory of mind and individual prediction accuracy rates (ρ varies between 0.04 and 0.24, all *p* > 0.117). This result stands in line with the previous evidence reported by Sylwester et al. (2012).

^9^At the time when our experiments were run, the usual exchange rate oscillated around 1 euro = 130 yen.

^10^For a perfect ability to discriminate between the two types of trustees, we would have *p*_*R*_ = 1 and *p*_*DR*_ = 0.

^11^This specification overcomes the usual caveats of using OLS for binary choice data. First, our specification with cluster-robust variance-covariance matrix is also heteroscedasticity-robust. Second, the forecasting issue (i.e., predicted probabilities going beyond the [0; 1] range) does not arise for binary explanatory variables: here, an estimated coefficient simply boils down to the respective choice proportion in a given experimental condition.

^12^To provide further statistical support for this result, we run additional analyses based on paired *t*-test. For each subject, we calculate the rate of prediction *Roll* for untrustworthy target agents, and then compare it to analogous rate calculated for the trustworthy ones. In all conditions other than VIDLO conducted in Japan, we find Bayes factor *BF*_10_ between 0.15 and 0.45 for a two-sided test, clearly testifying against the alternative hypothesis of a difference between the two rates. For the remaining condition, *BF*_10_ = 2.23, thus yielding support (although not overwhelming) for the alternative hypothesis of different rates. Repeating the same exercise for standard (i.e., non-Bayesian) *t*-test yields *p*-values and conclusions in line with those reported in Table 3.

^13^The three facial measurements have been obtained from the mugshot pictures used in the PHOTO treatment. For computation, we followed standard procedures adopted from Van Leeuwen et al. (2018) and summarized in Appendix B. See Stirrat and Perrett (2010) and Rodríguez-Ruiz et al. (2019) for a further discussion on the potential role of these facial characteristics in cooperation detection.

^14^Estimated coefficients from a logistic regression give comparable results. The main advantage of using OLS instead of a non-linear model is that in the latter, the only meaningful way to quantitatively interpret the estimated coefficients is by computing marginal effects. However, the use of marginal effects becomes problematic in the presence of interactions terms. The literature does not provide a clear-cut solution to this issue (see Ai and Norton, 2003; Greene, 2010). Since the statistical testing of interactions is central to the exercise reported in Table 4, we favor OLS (which allows us to easily operationalize interaction terms in the model) over a non-linear specification.

^15^For the French sample, we test the significance of coefficients β_1_ (PHOTO), β_1_ + β_4_ (VIDNE), β_1_ + β_5_ (VIDLO), neither of which is found to be significant (*p* = 0.363, *p* = 0.231, *p* = 0.740, respectively). For the Japanese data, the corresponding tests involve coefficients β_1_ + γ_1_ (*p* = 0.171), β_1_ + β_4_ + γ_1_ + γ_4_ (*p* = 0.145), β_1_ + β_5_ + γ_1_ + γ_5_ (*p* = 0.018).

^16^Two-sided ranksum test does not detect significant differences in facial masculinity (*p* = 0.959), asymmetry (*p* = 0.520), or width-to-height ratio (*p* = 0.382) between those that *Roll* (*N* = 14) and those that do not (*N* = 12). Moreover, both females and males choose to *Roll* with the same frequency (in 7 out of 13 cases); χ^2^ test yields *p* = 1.000.

^17^As shown in Table 4, coefficients β_6_ and β_9_ are positive and significant. This suggests that, *ceteris paribus*, higher facial masculinity, as well as being a female, increases the likelihood of being perceived as trustworthy person by a French subject. Insignificance of coefficients β_7_ and β_8_, in turn, suggests that there is no statistical association between being perceived as a trustworthy person and one's facial asymmetry or width-to-height ratio. The same results hold for the Japanese sample: coefficients β_*i*_ + γ_*i*_ are found to be positive and significant for *i* = 6 and *i* = 9 (both *p* < 0.001), but not for *i* = 7 (*p* = 0.489) and *i* = 8 (*p* = 0.057). Finally, a joint test of *H*_0_ : γ_6_ = γ_7_ = γ_8_ = γ_9_ = 0 does not reject the joint nullity of the differences between the respective coefficients across the two samples (*p* = 0.434).

^18^In Experiment 1 reported in Zylbersztejn et al. (2020), there are three conditions: neutral mugshot pictures (analogous to the PHOTO treatment used herein), neutral videos and loaded videos (analogous to VIDNE and VIDLO used herein, with one key difference: the sound is on, so that the subjects not only watch, but also listen to the target player's statement). Compared to the present experiment, the stimuli in that experiment are provided in a slightly different manner: the total set of stimuli consists of 41 items (including the 26 stimuli employed herein), and each subject inspects a randomly drawn sequence of 20 items. Focusing on the subset of the 26 target players that are common for both experiments, in Zylbersztejn et al. (2020) each item is shown to 21 subjects on average (range: 15–30 for pictures, 16–28 for both types of videos), while in the present experiment each subject inspects all 26 items. We believe that these differences do not distort subjects' predictions, so that the observations coming from the two sources remain comparable. Exploiting the data from the PHOTO condition (in which the stimuli contain the same information in both experiments), we compare the rates of prediction *Roll* for each of the 26 items registered in the present experiment to those from Zylbersztejn et al. (2020); signrank test yields *p* = 0.354. The same exercise for the VIDNE condition—in which neutral video recordings are muted in the present experiment, and contain the target player's voice in Zylbersztejn et al. (2020)—yields *p* = 0.525. This, in turn, corroborates the previous finding from Vogt et al. (2013) that hearing a stranger's voice in a neutral context does not *per se* affect the perception of that person's cooperativeness.

^19^The respective likelihoods are 69% (*N* = 16) and 30% (*N* = 10). χ^2^ test yields *p* = 0.054. Like Charness and Dufwenberg (2006), we define a promise as a statement of intent to *Roll*. Note that, as raised by Houser and Xiao (2011), the *ex post* interpretation of free-form messages is a major methodological challenge for the experimenter. The literature still lacks a common consensus on whether this should involve content analysis carried out by the experimenter (Charness and Dufwenberg, 2006), by independent coders (He et al., 2017), through an incentivized coordination game (Houser and Xiao, 2011), or by asking the subjects for their own interpretation (Servátka et al., 2011). Our classification method echoes the recent study by Schwartz et al. (2019). All statements were classified as promises or no-promises by two independent coders. The first coder classified the content of messages while preparing the transcripts of the trustees' statements. Then, another coder received a complete list of transcripts and independently classified each of them. Ties were broken by one of the authors.

^20^We note that implementing VIDLO_SOUND in the Japanese sample does not seem as a meaningful exercise due to a high degree of uncertainty as of the extent to which these subjects comprehend the verbal content of an improvised statement in French. Although their skills in foreign languages may be insufficient for understanding everything, they may nonetheless comprehend (or believe to be understanding) a part of this content (e.g, single words or sentences). This leaves an important degree of uncontrolled variation related to what a Japanese subject could potentially understand, how much, and how well, thus rendering the overall results hard to interpret.

^21^See Todorov et al. (2015b) for a systematic review of the empirical evidence on social attribution from faces.
