# Supplementary material for: Predicting Trustworthiness Across Cultures: An Experiment
Source: Front Psychol. 2021 Sep 28;12:727550. doi: 10.3389/fpsyg.2021.727550 (PMC8507433; doi:10.3389/fpsyg.2021.727550)
Supplement: Supplementary file 1 [file Presentation_1.pdf]

Predicting trustworthiness across cultures: An experiment
Supplementary material

Adam Zylbersztejn* Zakaria Babutsidze† Nobuyuki Hanaki‡

September 7, 2021

*Univ Lyon 2, Université Lumière Lyon 2, GATE L-SE UMR 5824, 69130 Ecully, France; research fellow at Vistula University Warsaw (AFiBV), Warsaw, Poland

†SKEMA Business School, Université Côte d'Azur (GREDEG) and OFCE, Sciences Po Paris

‡Institute of Social and Economic Research, Osaka University

A Experimental instructions

This appendix provides details of the implementation and instructions used in the hidden action game experiment of Babutsidze et al. (2021), as well as the instructions used in the present study.

A.1 Implementation of Babutsidze et al. (2021)

Each experimental session involves 6 trustors (referred to as player As in the experimental instructions) and 6 trustees (referred to as player Bs). All trustors remain in one room during the whole experiment. They are seated in a single row, isolated one from another by separators, and not allowed to talk. The space in front of them is left open and used by a trustee to make a brief statement. Trustees enter the room one by one, so that trustors play six rounds of the game (which is common knowledge). Each time, trustee faces the center of trustors' row, and all trustors have a clear view on the speaker. Trustee also has a clear, unobstructed view on all six trustors. After making a statement, trustee is invited to a separate room where s/he privately decides whether to *Roll* a die or not. Then, s/he is asked to leave the laboratory and wait outside until the end of the experiment. At the same time, each trustor makes a decision whether to go *In* or stay *Out*. All decisions are made on a sheet of paper, which is then put in an envelope, sealed, and collected by the laboratory staff after each round. In addition, once trustee has made a decision and left the separate room, a laboratory staff member rolls a die in private and marks the outcome on trustee's sealed envelope. At the end of the experiment, trustors and Bs are randomly and anonymously matched in pairs. The outcome of the game for each pair is based on the payoff structure described in Figure **??** and defined by the decision made by trustor after trustee's statement, as well as the decision made by trustee in a private room had the trustor chosen to go *In*. For the trustee's decision to *Roll*, the outcome of the die roll is also taken into account.

For the sake of logistics and efficient time management, trustees arrive 30 minutes prior to trustors. First, they are asked to take up several computerized tasks that measure their preferences and characteristics. Then, they are all led to a waiting room. To avoid any communication or subjects overhearing what others are saying or doing, each participant is seated in a separate cubicle, puts on a headphone and listens to a classical music until further notice. Then, they are taken one by one to a separate room for a mugshot picture and a short, standardized video recording.[1]

Then, each subject is seated back in his cubicle with headphones on and listens to an audio file containing the experimental instructions (paper version is also provided). There is a brief comprehension quiz assisted by a laboratory staff member. Finally, he receives additional paper instructions about the upcoming statement in front of trustors, as well as a pen and an empty

[1]Like in Van Leeuwen et al. (2018), subjects are asked to read neutral content (a short extract from a printer instruction manual) and keep a neutral face expression. The recording takes about 30 seconds.

sheet of paper, and is given approximately two minutes to prepare his message.[2] After that, a trustee is invited to trustors' room where he delivers a statement, leaves for another room, and the game proceeds to the decision-making stage. The average duration of a message is 26.39 seconds (SD 2.09). Trustees' statements are recorded using a small, non-intrusive video camera set up in the middle of trustors' row, right in front or trustees' zone, so that the perspective in the video camera recording resembles the one of a trustor. The camera is always adjusted to the height of trustee (so as to capture head, shoulders, and thorax), and to the luminosity in the room. The sake of the quality of the video recordings, the background in trustees' zone is covered with light canvas. While making a statement, each trustee also has a portable microphone attached below their face. The distance between trustors and a trustee is set to 2.50 meters.

Upon their arrival to the laboratory, trustors also take up the set of preliminary questionnaires. Then, they receive and read paper instructions for the experimental game, and finally they fill in a short comprehension quiz. A laboratory staff member then reads aloud all the questions from the quiz along with the corrects answers, and answers any remaining questions. Finally, trustors are asked to wait for the arrival of the first trustee.

There are 7 sessions. However, one trustee in session 6 decided to quit after the preliminary measurements and before receiving the instructions of the hidden action game, and was replaced by a research assistant unknown to trustors. To avoid any contamination of trustors' behavior, that research assistant acted as trustee in the final round of the experimental game. The data from that round were dismissed and our dataset from that session only covers 5 trustees, and thus 41 trustees in total.

A.2 Instructions used in Babutsidze et al. (2021)

A.2.1 Preliminary instructions given to all subjects

You are about to take part in an experiment in which you can earn money. The amount of your gains will depend on your decisions, as well as on the decisions made by other participants. In addition, you will receive a fixed fee of [*5 for player As, 10 for player Bs*] EUR for completing the experiment. Your total earnings will be paid privately in cash at the end of the experiment.

The experiment consists of several parts. Each part will involve tasks the rules of which will be explained to you in due time. It is crucial that you understand and obey the rules of this experiment. Violation of these rules might result in an exclusion from the experiment and all payments. Please raise your hand whenever you have questions or need assistance.

[2] Those additional instructions remind the subject about his role in the game; emphasize the fact that the message may affect trustors' decisions and, consequently, the subject's gain from the experiment; instruct the subject to avoid making a visual or verbal contact with the experimenter, to aim at communicating with all trustors, and not to introduce oneself or give any details about one's own identity.

All the information you provide, as well as the amount of your gains from this experiment, will remain strictly confidential and anonymous.

We would now like to ask you to answer a series of preliminary questions. You will answer these questions using the interface on your computer screen. Some of these questions will generate monetary gains. These gains will be determined and added to your overall earnings at the end of the experiment.

Note: the following instructions were only given for the preliminary recordings to participants acting as player Bs.

Now, we would like to take a picture and video recoding of you.

First, you will be asked to stand by the wall and look into the camera. Please, try to keep a neutral facial expression.

Second, you will be asked to read aloud the content display on the screen in front of you. While reading, you will be video recorded.

All pictures and video recordings produced during this experiment will only serve strictly scientific purposes of this research project. They may be used in other experimental sessions related to this research project.

A.2.2 Instructions for the hidden action game

Rules of the game

You will now play a game with monetary stakes. The rules of the game are as follows.

The game is played by two players: player A and player B. Each player must choose between two possible actions. Player A chooses between actions "Left" and "Right". Player B chooses whether she want a six-sided die to be rolled (action "Roll") or not (action "Don't roll").

You will play the role of player [*A for player As, B for player Bs*]

Each players' payoff depends on the actions chosen by herself as well as the other player:

- if player A chooses "Left", then regardless of player Bs' choice:
  - player A's payoff is 5 EUR and player B's payoff is 5 EUR;
- if player A chooses "Right" and player B chooses "Don't roll":
  - player A's payoff is 0 EUR and player B's payoff is 14 EUR;
- if player A chooses "Right" and player B chooses "Roll":
  - if the number of on the die is between 1 and 5, then player A's payoff is 12 EUR and player B's payoff is 10 EUR;
  - if the number of on the die is 6, then player A's payoff is 0 EUR and player B's payoff is 10 EUR;

How the game proceeds

The game will consist of six identical rounds.
At the beginning of a round, one player B is asked to enter the room in which there are six players As. Player As are separated one from another and are not allowed to talk.
Player B is then placed in front of player As and remains silent. Then, player B is allowed to talk for no longer than 20 seconds, and then asked to leave player As' room. While talking, player B is video recorded and should look straight into the camera.
Once player B leaves player As' room:

- player B makes a decision in a separate room. Player B privately and individually indicates her decision (either "Roll" or "Don't roll") on a separate answer sheet, puts it in an envelope and seals the envelope. The experimenter collects the envelope and player B leaves the room. Then, the experimenter privately rolls a six-sided die and marks the result on the envelope (without opening it). The outcome of the die roll will only be taken into account if player A's decision is "Right" and player B's decision is "Roll".

- each player A privately and individually indicates her decision (either "Left" or "Right") on a separate answer sheet, puts it in an envelope and seals the envelope. Then, all the envelopes are collected by the experimenter. Player As are either asked to remain silent and await the next player B, or informed that the experiment is over and given further instructions about their payment.

No envelope will be opened before the end of the experiment.
At the end of the experiment, each player A is anonymously and randomly matched with one player B. The outcome of the game for each pair of players is determined by the decisions made by both playes (and also by the outcome of the die roll if the decisions in a pair are "Right" and "Roll") in the round in which the player B was in player As' room. Players are only informed about their personal payoffs, and not about the payoffs of or the decisions made by other players, or about the outcome of the die roll.

Additional information

Note that this set of instructions is provided to and read by each player A and each player B. Furthermore, player Bs cannot communicate between themselves at any point of the experiment. The same applies to the communication between player As.

You will play the role of player [*A for player As, B for player Bs*]

A.3 Experimental instructions used in the present experiment

You are about to take part in an experiment in which you can earn money. The amount of your gains will depend on your decisions, as well as on the decisions made by other participants. In addition, you will receive a fixed fee of 5 EUR for completing the experiment. Your total earnings will be paid privately in cash at the end of the experiment.

The experiment consists of several parts. Each part will involve tasks the rules of which will be explained to you in due time. It is crucial that you understand and obey the rules of this experiment. Violation of these rules might result in an exclusion from the experiment and all payments. Please raise your hand whenever you have questions or need assistance.

All the information you provide, as well as the amount of your gains from this experiment, will remain strictly confidential and anonymous.

We would now like to ask you to answer a series of preliminary questions. You will answer these questions using the interface on your computer screen. Some of these questions will generate monetary gains. These gains will be determined and added to your overall earnings at the end of the experiment.

Note: Below, the parts of instructions that are distinct for each treatment are marked with "(treatment's name:)". Other parts are common to all three treatments.

(PHOTO:) In this part of the experiment, you will see a series of pictures of people.

(VIDLO and VIDNE:) In this part of the experiment, you will watch a series of video recordings. In each recording, you will see a person making a short statement.

You will be asked to predict the decisions those people previously made in another experiment (the details of which are described below). Your final gain will depend on the accuracy of your predictions.

The previous experiment

In each session, two groups of participants (six players A and six players B) were installed in two different rooms. Participants in each room could not communicate with each other. They all received instructions explaining the rules of the experiment they were about to participate in. Players were informed that their decisions and earnings would remain private and anonymous, and would never be disclosed to other participants.

Each session was organized as follows:

1. One by one, player Bs entered the room in which players A were sitting. Then, each player B made a short speech in front of player As. Before entering the room, each player B was

give a couple of minutes to prepare the statement. Each player B was also informed that his statement could affect player As' decisions and, consequently, his own gain in the experiment. (VIDLO:) **All the statements have been recorded, and you will be watching some of them**.

2. After his speech, player B left player As' room, and entered an empty room.

3. After player B's departure, each player A made a decision ("Left" or"Right") in private and individually. At the same time, player B made a decision ("Roll" or "Don't roll" a die) in privately and individually.

4. Thereafter, player B left the room and waited outside the laboratory until the end of the experiment. Meanwhile, a new player B was entering the players' room A to make a speech. The experiment ended when all the players had completed their task.

At the end of the experiment, each player A was anonymously and randomly matched with a player B. The outcome of the game for each pair of players was determined by the decisions made by both players following player B's speech:

- if player A chose "Left", then regardless of player Bs' choice:
  - player A's payoff was 5 EUR and player B's payoff was 5 EUR;
- if player A chose "Right" and player B chose "Don't roll":
  - player A's payoff was 0 EUR and player B's payoff was 14 EUR;
- if player A chose "Right" and player B chose "Roll":
  - if the number of on the die was between 1 and 5, then player A's payoff was 12 EUR and player B's payoff was 10 EUR;
  - if the number of on the die was 6, then player A's payoff was 0 EUR and player B's payoff was 10 EUR;

Your role

(PHOTO:)

This experiment consists of **26 rounds**. At the beginning of each round, you **will see a picture.**

Each picture presents a person in the role of player B from the previous experiment, as described above. The picture was taken privately and independently of the previous experiment.

Then, you will be asked to predict if the player B from the picture decided to roll a die in the previous experiment. Your gain will depend on the accuracy of your prediction: you will earn 10 euros for correct prediction and 2 euros for an incorrect one.

(VIDNE:)

This experiment consists of **26 rounds**. At the beginning of each round, you **will watch a short video recording (with the sound off).**

Each recording presents a person in the role of player B from the previous experiment, as described above. The recording was made privately and independently of the previous experiment.

Then, you will be asked to predict if the player B from the picture decided to roll a die in the previous experiment. Your gain will depend on the accuracy of your prediction: you will earn 10 euros for correct prediction and 2 euros for an incorrect one.

(VIDLO:)

This experiment consists of **26 rounds**. At the beginning of each round, you **will watch a short video recording (with the sound off).**

Each recording presents the statement made by a player B in front of player As during the previous experiment, as described above.

Then, you will be asked to predict if the player B from the recording decided to roll a die in the previous experiment. Your gain will depend on the accuracy of your prediction: you will earn 10 euros for a correct prediction and 2 euros for an incorrect one.

At the end of the experiment, two rounds will be drawn at random. Your final gain will correspond to the predictions you have made in those two rounds.

B Facial measurements

To obtain facial measures of the target players, we adopted the procedures described in Appendix A3 in Van Leeuwen et al. (2018) and applied them to the mugshot pictures gathered for our PHOTO treatment. Following their method, we first used the Image J software to mark 19 distinct points on each face, and then calculate 11 distances. Then, this information was used to compute three facial measures, as explained below.

Facial masculinity. This measure consists of four di erent ratios that have found to be sexually dimorphic. These four ratios are cheekbone prominence, which takes the ratio between the facial width at the cheekbones and at the jaws, the ratio between the jaw height and the lower face height, the ratio between the lower face height and the face height, and the ratio between facial width at the cheekbones and lower face height. Each of the four ratios is converted to a z-score and these z-scores are nally summed to one score.

Facial asymmetry. First, we compute the absolute differences between the left and right distance from a midline on 6 different points. The x-coordinate of the midline is computed by the midpoint of the distance between the pupils. Then, we compute the absolute differences for the inner eye corners, outer eye corners, cheekbones, nose, mouth and the jaw. To account for possible differences in distance from the camera, each of the absolute differences is normalized by dividing it by the inter-pupillary distance. Each of the absolute differences is converted to a z-score and summed up to one asymmetry score.

Width-to-height ratio. This is the ratio between the bizygomatic width and the upper face height (i.e., the distance between highest point of the eyelids and the top of the mouth).

References

Babutsidze, Z., N. Hanaki, and A. Zylbersztejn (2021): "Nonverbal content and trust: An experiment on digital communication," Economic Inquiry, forthcoming.

Van Leeuwen, B., C. N. Noussair, T. Offerman, S. Suetens, M. Van Veelen, and J. Van De Ven (2018): "Predictably angry – facial cues provide a credible signal of destructive behavior," Management Science, 64, 3352–3364.
